# Rhesus Macaque CODEX Multiplexed Immunohistochemistry Panel for Studying Immune Responses During Ebola Infection

**DOI:** 10.3389/fimmu.2021.729845

**Published:** 2021-12-06

**Authors:** Sizun Jiang, Nilanjan Mukherjee, Richard S. Bennett, Han Chen, James Logue, Bonnie Dighero-Kemp, Jonathan R. Kurtz, Ricky Adams, Darci Phillips, Christian M. Schürch, Yury Goltsev, John W. Hickey, Erin F. McCaffrey, Alea Delmastro, Pauline Chu, J. Rachel Reader, Rebekah I. Keesler, José A. Galván, Inti Zlobec, Koen K. A. Van Rompay, David X. Liu, Lisa E. Hensley, Garry P. Nolan, David R. McIlwain

**Affiliations:** ^1^ Department of Pathology, Stanford University School of Medicine, Stanford, CA, United States; ^2^ Center for Virology and Vaccine Research, Beth Israel Deaconess Medical Center, Boston, MA, United States; ^3^ Integrated Research Facility, Division of Clinical Research, National Institute of Allergy and Infectious Diseases, National Institutes of Health, Frederick, MD, United States; ^4^ Department of Pathology and Neuropathology, University Hospital and Comprehensive Cancer Center Tübingen, Tübingen, Germany; ^5^ California National Primate Research Center, University of California, Davis, CA, United States; ^6^ Institute of Pathology, University of Bern, Bern, Switzerland

**Keywords:** codex, EBOV (Ebola virus), rhesus macaque (Macaca mulatta tcheliensis), NHP (non-human primate), Spatial biology, multiplexed immunofluorescencence and immunohistochemistry

## Abstract

Non-human primate (NHP) animal models are an integral part of the drug research and development process. For some biothreat pathogens, animal model challenge studies may offer the only possibility to evaluate medical countermeasure efficacy. A thorough understanding of host immune responses in such NHP models is therefore vital. However, applying antibody-based immune characterization techniques to NHP models requires extensive reagent development for species compatibility. In the case of studies involving high consequence pathogens, further optimization for use of inactivated samples may be required. Here, we describe the first optimized CO-Detection by indEXing (CODEX) multiplexed tissue imaging antibody panel for deep profiling of spatially resolved single-cell immune responses in rhesus macaques. This 21-marker panel is composed of a set of 18 antibodies that stratify major immune cell types along with a set three Ebola virus (EBOV)-specific antibodies. We validated these two sets of markers using immunohistochemistry and CODEX in fully inactivated Formalin-Fixed Paraffin-Embedded (FFPE) tissues from mock and EBOV challenged macaques respectively and provide an efficient framework for orthogonal validation of multiple antibody clones using CODEX multiplexed tissue imaging. We also provide the antibody clones and oligonucleotide tag sequences as a valuable resource for other researchers to recreate this reagent set for future studies of tissue immune responses to EBOV infection and other diseases.

## Introduction

Animal models are vital for understanding disease pathogenesis as well as the development and evaluation of therapeutics. When human studies are not feasible, regulatory decision-making for medical countermeasures (MCMs) may rely entirely on outcomes from animal models, as has been the case for some potential bioterror threat pathogens (1). Non-human primate (NHP) models with similar phylogenetics and physiology to humans remain the only option for modeling many host-restricted viral infectious diseases (2). A thorough understanding of host immune responses in such NHP models is therefore imperative.

Since its discovery in 1976, Ebola virus (EBOV) has accounted for more than 33,000 documented infections and nearly 15,000 deaths, with a case fatality ratio of approximately 44% (3). Rhesus macaque lethal infection models provide a unique opportunity to understand EBOV pathogenesis and test MCMs against this biothreat (4). However, studying EBOV tissue pathogenesis in this context is challenging due to the difficulties of working inside maximum containment, the incompatibility of many assays with inactivated samples, and the scarcity of NHP-specific reagents.

Not surprisingly, a comprehensive understanding of tissue-level immune responses to EBOV infection is still lacking. To date, our ability to examine the complex host immune responses to viral infections *in situ* has been hindered by technical barriers that allow only a limited number of markers to be simultaneously examined on cells using traditional fluorescent microscopy. Recently developed multiplexed imaging techniques offer great promise for more precise profiling of the spatial biology of immune responses to EBOV (5). A new method named CO-Detection by indEXing (CODEX) (6) bypasses traditional fluorescent microscopy limits by simultaneous staining of tissue samples with a cocktail of DNA-indexed antibodies followed by iterative steps of hybridization with complementary, fluorescently labeled probes for imaging. This results in the generation of images with up to 60 parameters, enabling the extraction of high-parameter, spatially-resolved single-cell data from solid tissue samples.

An important consideration of antibody-based immune profiling techniques such as CODEX is the significant initial efforts required to screen and optimize reagents for compatibility with both NHP models (7–10) and inactivation protocols required to safely work with samples outside of maximum containment.

We describe here the validation workflow and establishment of the first CODEX antibody panel specifically designed for use with inactivated, archival rhesus macaque tissue samples. This 21-marker panel includes 18 antibodies for the identification of major immune cell types, along with three EBOV-specific antibodies. Our resource also includes a list of all 75 antibodies tested and manufacturer-reported cross-reactivity. This panel will enable future studies characterizing the immune cell infiltrates and spatial organization of host-EBOV tissue interactions *in situ* and signifies a key starting point for work extending into other NHP disease models.

## Results

### Overview of Experimental Workflow

**Figure 1:**
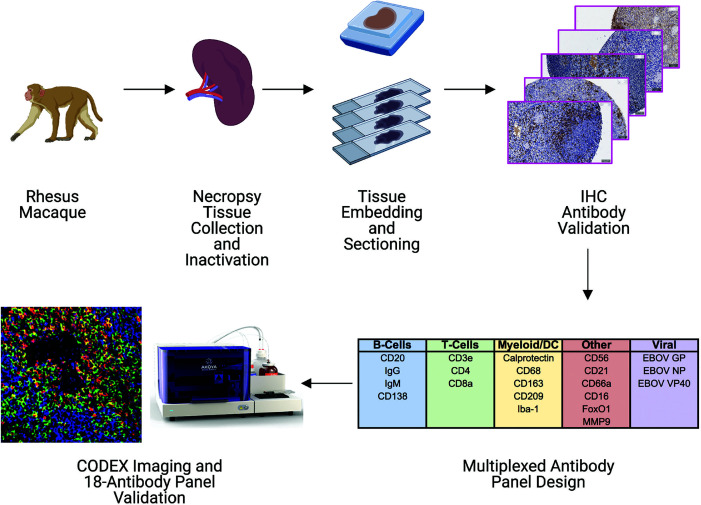
Pipeline for building a CODEX antibody panel for studying tissue immune responses during EBOV infection in rhesus macaques. Spleen tissues were collected from control and EBOV challenged rhesus macaques, inactivated by fixation, embedded in paraffin blocks, and sectioned. A panel of antibodies targeting host immune cells and EBOV proteins was tested by immunohistochemistry (IHC) to determine compatibility with epitopes following inactivation. Antibodies with acceptable staining performance by IHC were conjugated to unique DNA oligonucleotide tags and pooled to create a 21-plex CODEX antibody panel. CODEX antibody panel validation was accomplished by staining tissues with the entire antibody panel cocktail and examining orthogonal staining patterns of antibody channels after imaging.

In this study, we designed and implemented a 21-marker CODEX antibody panel compatible with inactivated FFPE rhesus macaque tissue samples to enable future studies into EBOV pathogenesis. Our proof-of-concept work uses tissues from a previously reported investigation of EBOV-challenged and healthy control rhesus macaques (4, 11). Spleen tissues collected at necropsy were inactivated and processed into FFPE blocks. Tissues were subsequently sectioned and immunohistochemistry (IHC) antibody validation performed against several immune phenotyping and virus-specific markers. Markers that passed our IHC antibody validation were then incorporated into a multiplexed panel for downstream CODEX imaging and processing **(** **Figure 1** **)**.

### Host Antibody Validation in Healthy Lymphoid Tissues

**Figure 2:**
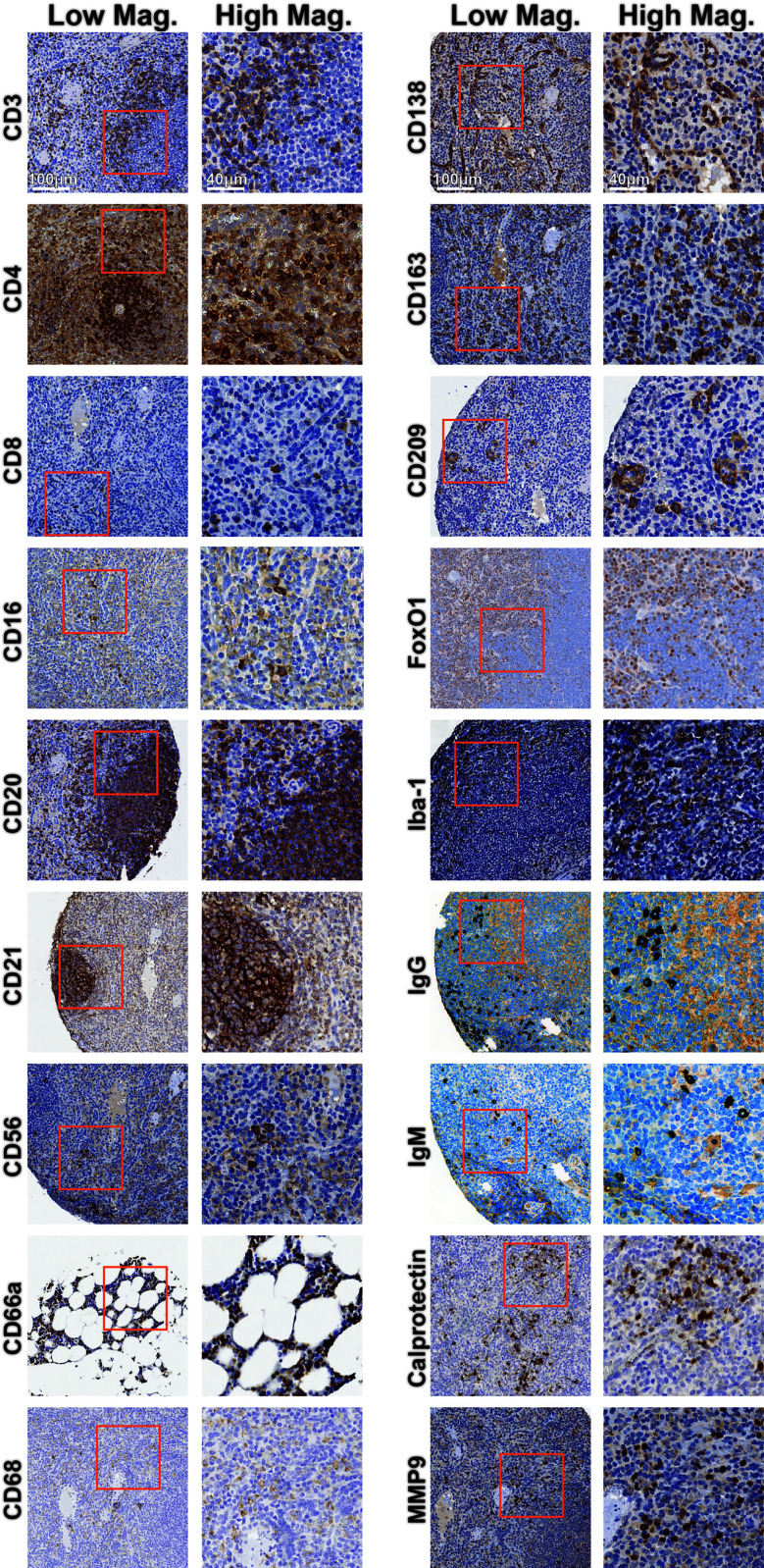
Immunohistochemistry validation of antibodies targeting rhesus immune tissues. Representative low magnification (left) and high magnification (right) IHC images for indicated markers. Inlaid orange boxes on low magnification images indicate the magnified region.

The initial host antibody marker selection was based on prior knowledge and antibody clones described in previous studies (12–19). Various factors, such as the time between tissue collection and fixation, duration of fixation, processing into paraffin blocks, and storage conditions can affect tissue integrity and immunohistochemical analysis (20). We rigorously titrated each of the 18 immune-specific antibodies by IHC using healthy control tissues treated with the same tissue fixation and processing conditions suitable for EBOV inactivation and the same heat-induced epitope retrieval conditions intended for the subsequent CODEX experiments **(** **Figure 2** and **Supplementary Table 1** **)**. Staining of spleen and bone marrow tissues resulted in consistent staining patterns and expected spatial distributions of marker positive cells in comparison to The Human Protein Atlas pathology data (21), as well as with our internal human tissue controls (**Supplementary Figure 1**). For example, CD20 was found appropriately within B cell follicles, while FoxO1 was found in the nucleus of lymphocytes and macrophages **(** **Figure 2**, middle left, middle right). This laid a foundation for subsequent multiplexed orthogonal validation of markers by CODEX.

Reagent compatibility with multiplexed imaging methods, such as multiplexed ion beam imaging (MIBI), imaging mass cytometry (IMC), and CODEX, all require these antibodies to 1) be available in a carrier-free purified form, 2) retain its antigen-binding capabilities after labeling with metal conjugates or barcoded oligonucleotides, and 3) perform in concert in a cocktail containing antibodies against different targets (5). Each antibody clone that passed initial validation by IHC was subsequently conjugated to a unique indexed DNA oligonucleotide tag (**Supplementary Table 1**) **(** 22) and tested by CODEX imaging. Only antibodies that passed our initial IHC screening and CODEX validation pipeline are described in this report (see *Discussion* ).

**Figure 3:**
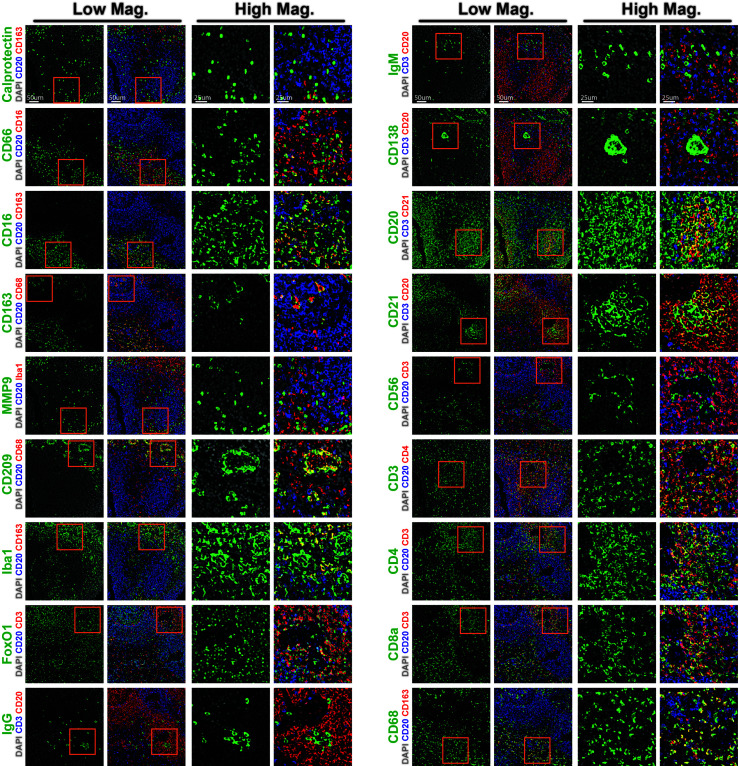
CODEX validation of antibodies targeting rhesus immune tissues. Representative low magnification (left) and high magnification (right) CODEX images for indicated markers (green). Markers are shown relative to nuclear stain only (DAPI, grey, left), or overlayed with co-staining (red) and counterstaining (blue) markers to demonstrate specificity (right). Inlaid orange boxes on low magnification images indicate the magnified region.

A key aspect of antibody-based assays is the need for thorough reagent validation (23). A fundamental strength of performing this on a multiplexed imaging platform, such as CODEX in this study, is the ability to orthogonally validate antibody reagents against each other. To exemplify this, we generated graphical three-marker-overlays of CODEX images from this study **(** **Figure 3** **)**. These overlays were designed to highlight the key marker being investigated in green, an overlapping marker in red, and a mutually exclusive marker in blue. For instance, we confirmed the specificity of the antibody against calprotectin, an intracytoplasmic marker that is found predominantly in monocytes and macrophages, by observing overlap with another macrophage marker, CD163, but mutually exclusive staining patterns with the B cell-specific marker CD20 (**Figure 3**, top lef). Similarly, we confirmed strong overlap between the T cell marker CD3 with either CD4 or CD8, but not with CD20, and also the macrophage marker CD68 with CD163, but not with CD20 (**Figure 3**, bottom right). We thusly confirmed the specificity of neutrophil markers (CD66 and CD16), macrophage markers (CD68, CD163, MMP9, CD209, FoxO1, and Iba1), B cell markers (CD20, CD21, CD138, IgG, and IgM), natural killer cell markers (CD16 and CD56), follicular dendritic cell marker (CD21), dendritic cell marker (CD209) and T cell markers (CD3, CD4, CD8a and FoxO1) (**Figure 3**). These results highlight the importance of cross-validating antibody reagents against co-expressed or mutually-expressed markers and provide an efficient framework to validate antibody reagents using CODEX multiplexed imaging.

### Differential Expression of Cell Type-Specific Markers Revealed by CODEX Multiplexed Imaging

**Figure 4:**
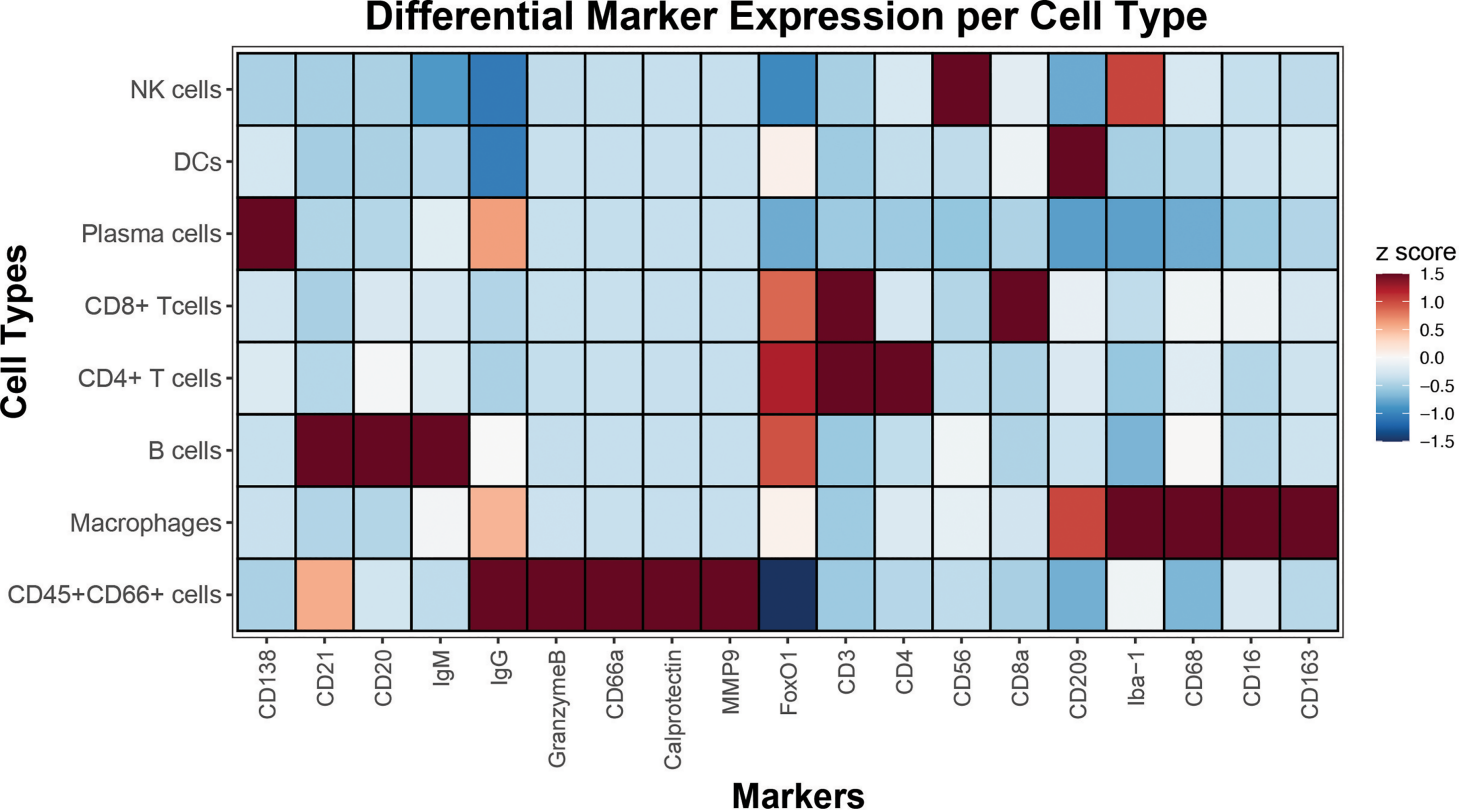
Cell type-specific marker expressions revealed by CODEX. Single-cell data extracted from segmented CODEX images of spleen from this study was used to identify major immune cell populations by gating. A heatmap displays rows signifying cell types, and columns indicative of their corresponding marker expressions. Median z scores and their corresponding color maps are shown in the key on the right.

High dimensional imaging with the 18 lineage-specific and functional markers allowed further delineation of cell types and their unique expression patterns. This confirmed the appropriate presence of phenotypic markers CD56 in NK cells, CD209 in DCs, CD138 and some IgG in Plasma cells, CD3 and CD8 in CD8 T cells, CD3 and CD4 in CD4 T cells, CD20, CD21 and IgM in B cells and CD209, Iba-1, CD68, CD16 and CD163 in macrophages (**Figure 4**). This approach orthogonally confirmed the staining specificity and distinct differential lineage-specific marker expression patterns as revealed by CODEX multiplexed imaging.

### EBOV-Specific Antibody Validation in EBOV-Challenged Lymphoid Tissues

**Figure 5:**
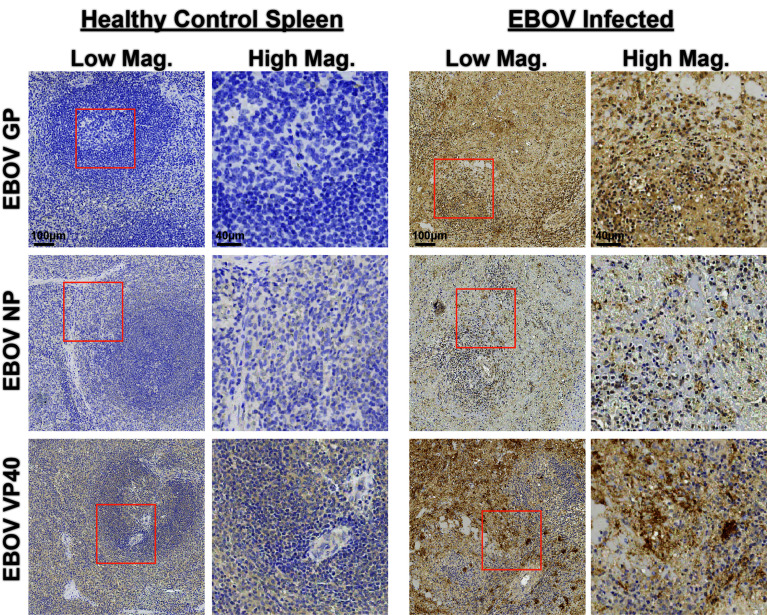
Immunohistochemistry validation of anti-EBOV protein antibodies. Representative low magnification (left, center right) and high magnification (center left, right) IHC images for indicated EBOV protein markers on healthy control spleen sections (left) or spleen sections from EBOV challenged animals (right). Inlaid orange boxes on low magnification images indicate the magnified region.

To enable future studies to dissect intricate viral tissue interactions, we next sought to extend our antibody validation to EBOV-specific reagents. We identified three antibodies from previous literature targeting the EBOV structural glycoprotein (GP), viral RNA encapsidation nucleoprotein (NP), and virion assembly protein and interferon antagonist VP40 (18, 19, 24). We first tested these antibodies using IHC on spleen collected from healthy controls and EBOV-infected NHPs (**Figure 5**). We confirmed their strong signal specificity for EBOV in infected spleen, as well as antibody performance in our standardized staining protocol.

**Figure 6:**
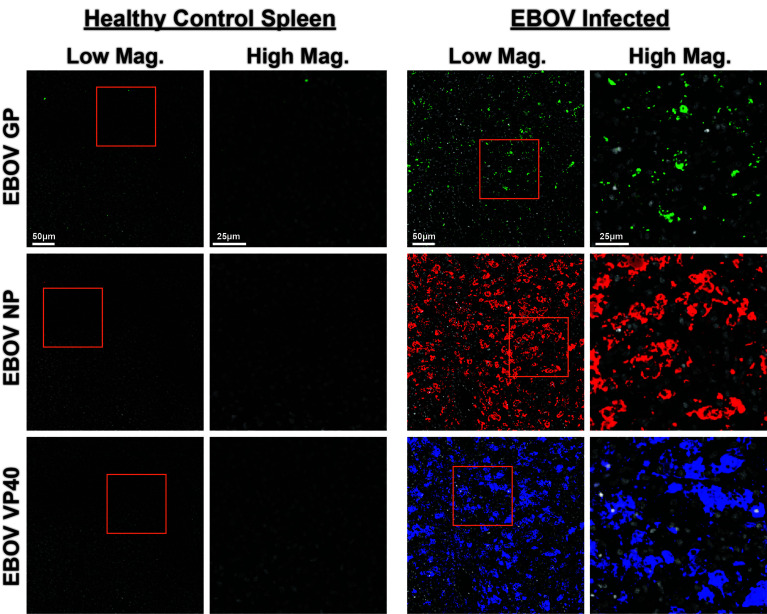
CODEX validation of anti-EBOV protein antibodies. Representative low magnification (left, center right) and high magnification (center left, right) CODEX images for indicated EBOV protein markers on healthy control spleen sections (left) or spleen sections from EBOV challenged animals (right). EBOV GP (green), EBOV NP (red), EBOV VP40 (blue). Inlaid orange boxes on low magnification images indicate the magnified region.

We next conjugated these anti-EBOV antibodies to unique indexed DNA oligonucleotide tags (**Supplementary Table 1**) and added them to the CODEX panel. Staining of EBOV-infected and healthy control spleens indicated anti-EBOV antibodies performed well in concert, with minimal background observed in healthy controls and specific signals confirmed in infected samples (**Figure 6**). These results indicate that the epitope binding capabilities of these EBOV-specific antibodies were retained even after DNA-oligonucleotide conjugation and perform robustly during multiplexed CODEX imaging.

## Discussion

In this study, we describe the implementation of the first optimized CODEX multiplexed tissue imaging panel for deep profiling of spatially resolved single-cell immune responses in inactivated, archival rhesus macaque tissues. This resource is paramount for future studies of host responses to EBOV, with broad applicability to other research topics. The 21-marker FFPE compatible panel includes 18 antibodies for the identification of major immune cell types along with three EBOV-specific antibodies. Host markers were validated by co-expression and orthogonal staining patterns of canonical targets used for immune cell type identification, while EBOV-specific antibodies were validated by the use of tissues from healthy control and EBOV-challenged animals.

Given that NHP models are critical tools to study and develop effective therapies for high consequence pathogens, the assembly of this CODEX antibody panel empowers future investigations into tissue-specific responses against biothreats in these models. We share below the unique set of challenges encountered in this study which we hope will allow a smooth transition for others in adopting antibody-based high-dimensional tissue imaging for NHPs.

First, commercial antibodies used for studying NHPs are generally limited to those raised against human epitopes and must be tested for compatibility with the specific NHP species in question. The IHC-based screening was used in this study as an initial step to establish the compatibility of antibody clones with rhesus macaque tissues. We also provide here all the antibodies tested in this study, including their manufacturer suggested cross-species compatibility and IHC-compatibilities (**Supplementary Table 2**). Eighteen of the seventy five antibodies tested passed our validation pipeline and eventually were compatible with CODEX staining. Our validation pipeline tested antibodies at only two uniform dilutions with epitope retrieval conditions identical to that used for CODEX staining. It is probable that some clones that failed our validation pipeline may indeed perform adequately under different tissue fixation conditions, retrieval conditions, or antibody dilutions.

Second, currently available high parametric protein imaging platforms, such as CODEX, MIBI, cycIF, IMC, and others (6, 12, 25–29) require complex instrumentation that would be difficult to operate inside a maximum containment environment (5). Therefore, we optimized the CODEX panel described in this study to be compatible with FFPE tissues resulting from the stringent inactivation protocols required to safely remove samples from maximum containment laboratories. FFPE tissues are advantageous for ease of storage and general accessibility of archival samples, However, they present certain difficulties relative to fresh-frozen tissues as fixation can alter the conformation of epitopes, precluding the binding of antibodies without the use of target-specific antigen retrieval steps. This is a greater challenge for CODEX where all the antibody clones in a single panel must be compatible with a single set of antigen retrieval conditions; we optimized the CODEX panel described in this study to be compatible with such constraints (i.e., target retrieval solution (pH9) at 97°C for 10 min).

Lastly, after clones are deemed to perform adequately with fixation and retrieval conditions by IHC, antibodies require conjugation to DNA oligonucleotides and further validation, since the mildly-reducing conjugation process can have a detrimental effect on the antibody performance. Issues can also arise with the oligonucleotide tags themselves. Maleimide-tagged oligonucleotides require deprotection using a Diels-Alder reaction prior to conjugation, and it is important that each batch of oligonucleotides is validated with a known antibody clone to ensure the fidelity of this deprotection process (22). The efficacy of antibody conjugation can be negatively impacted by multiple factors, such as the incompatibility of the antibody with the TCEP mild reduction for maleimide conjugation and the presence of contaminating proteins in solution. We, therefore, emphasize that antibodies must be purchased carrier-free when available or purified before oligonucleotide conjugation.

Given these challenges, the success rate for the inclusion of putative antibodies in this panel was low (24%), making the initial development of our optimized CODEX panel costly and time-consuming. However, once suitable clones and oligonucleotide sequences are identified, a panel is easy to reproduce and be expanded upon by other investigators. We are confident that this panel and the current availability of validated CODEX oligonucleotide channels (12, 22) will provide a core set of markers that other investigators can build upon.

In sum, we present here a rhesus macaque-specific CODEX panel that is immediately available to interrogate the spatial immune microenvironment in response to the progression of Ebola virus disease. Future studies will use this panel to examine the phenotype and abundance of different immune cell subsets responding to and participating directly in viral replication in tissues. Our long-term efforts will aim to not only understand how individual cell types respond to infection but also how cells functionally organize into neighborhoods to mount coordinated immune responses to pathogens.

## Materials and Methods

### Animal Study

Tissue samples were obtained from a previously reported EBOV Kikwit challenge study in rhesus macaques (4). That study was conducted at a National Institute of Allergy and Infectious Diseases (NIAID) facility under the approval of the NIAID Division of Clinical Research Animal Care and Use Committee strictly adhering to the Guide for the Care and Use of Laboratory Animals of the National Institute of Health, the Office of Animal Welfare, and the US Department of Agriculture. Water and food were available ad libitum. Animals were anesthetized prior to clinical procedures conducted by trained personnel under the supervision of veterinary staff. Animals were challenged with 1000 PFU EBOV/Kikwit in the left lateral triceps muscle diluted in a total volume of 1 mL at the study day 0 (4). Tissues described in the current study were collected during necropsy from unchallenged control animals NHP C1 and NHP C3 or from challenged animals NHP 7 (necropsied on study day 7 with viremia of 1.7E10 copies/mL) and animal NHP 8 (necropsied on study day 6 with viremia of 1.6E10 copies/mL) as previously described (4, 11). Additional healthy control rhesus tissues for initial antibody clone validation were obtained from the California National Primate Research Center (CNPRC), University of California, Davis. The CNPRC is accredited by the Association for Assessment and Accreditation of Laboratory Animal Care International (AAALAC). Animal care is performed in compliance with the 2011 Guide for the Care and Use of Laboratory Animals provided by the Institute for Laboratory Animal Research. Macaques were housed indoors in stainless steel cages (Lab Product, Inc.) whose sizing was scaled to the size of each animal, as per national standards, and were exposed to a 12-hour light/dark cycle, 64-84°F, and 30-70% room humidity. Animals had unrestricted access to water and received commercial chow (high protein diet, Ralston Purina Co.) and fresh produce supplements. Studies were approved by the Institutional Animal Care and Use Committee of the University of California, Davis.

### Tissue Processing and Sectioning

Tissue chunks up to 1cm^3^ in size from necropsied animals were inactivated in a 10% neutral buffered formalin (NBF) fixative at a ratio of at least 20 parts fixative to 1 part tissue (vol/vol) for a minimum of 72h. Complete replacement of 10% NBF fixative occurred prior to removal of any samples from containment. Fixed samples were embedded in paraffin blocks and subsequently cored and re-embedded into Next-generation Tissue Microarray (ngTMA) paraffin blocks. Regions of interest were annotated in Case Viewer software (3DHistech, Budapest, Hungary) on digitized hematoxylin and eosin (H&E)-stained sections. For each specimen, three representative tissue cores of 0.6 mm diameter were assembled into ngTMAs using a Grand Master automated tissue microarrayer (3DHistech). Blocks were cut in 4-µm thick sections using a Leica Reichert-Jung 2030 Biocut Manual Rotary Microtome onto frosted histology glass slides (12-550-15, Thermo Fisher) for IHC or Vectabond (SP-1800, Vector Laboratories) treated coverslips for CODEX (see ‘*Coverslip Preparation*’ below).

### Antibodies

Purified carrier-free antibodies were purchased from commercial suppliers. Clones and suppliers are listed in **Supplementary Table 1**.

### Immunohistochemistry

FFPE sections on glass slides were used for IHC. Slides were deparaffinized by baking at 70°C for at least 1 hour. Slides were then immersed in fresh xylene (X5-4, ThermoFisher) for 30 min (two separate containers, 15 min each). Next, slides were rehydrated in descending concentrations of ethanol (412811, Gold Shield) (twice in 100%, twice in 95%, once in 80%, once in 70%, twice in ddH2O; each step for 3 min). Slides were loaded into slide chambers containing 1X Target Retrieval Solution, pH9 (S236784-2, Agilent), and heat-induced antigen retrieval (HIER) was performed using a PT Link Pre-Treatment Module (Dako, Agilent) at 97°C for 10 min. After antigen retrieval, slide chambers were removed from the module and allowed to equilibrate to room temperature for 30 min. Tissue sections were then encircled on slides using a polyacrylamide gel pen (Bondic). The slides were washed twice with 1X TBS IHC wash buffer containing Tween20 (935B-09, Cell Marque) at room temperature for 5 minutes. Slides were blocked for 1 hour at room temperature using 100 µL of serum-free protein block (X090930-2, Agilent) to prevent nonspecific antibody binding. Antibodies were diluted in 100 µL antibody diluent (S080983-2, Agilent) (see **Supplementary Table 1** for concentrations), and sections were stained overnight in a sealed humidity chamber at 4°C on a shaker. After overnight staining, slides were washed twice with 1X TBS IHC wash buffer containing Tween20 for 5 minutes. Sections were covered with dual endogenous enzyme-blocking reagent (S200389-2, Agilent) for 5-10 min at room temperature, followed by two washes with 1X TBS IHC wash buffer containing Tween20 for 5 minutes each. Excess wash buffer was tapped off and 100 µL of EnVision+ Dual Link, Single Reagents (HRP Rabbit/Mouse) (K406311-2, Agilent) was added for 30 min at room temperature and then washed. Bound antibodies were visualized using the HRP/liquid DAB+ substrate chromogen system (K346711-2, Agilent) according to the manufacturer’s instructions. Sections were counterstained with hematoxylin (GHS116-500ML, Sigma). Stained IHC slides were digitally scanned using an Aperio AT2 Digital Whole Slide Scanner (Leica Biosystems) with images examined using Aperio ImageScope (version v.12.4.3.5008) and QuPath (version 0.2.3) software.

### Coverslip Preparation

For CODEX assays, square (22 x 22 mm) glass coverslips (72204-10, Electron Microscopy Sciences) were pre-treated with Vectabond (Vector Labs) according to the manufacturer’s instructions. Briefly, using glass beakers, coverslips were immersed in 100% acetone for 5 min and then incubated in a mixture of 7 mL Vectabond and 350 mL 100% acetone for 30 min. Coverslips were washed in 100% acetone for 30 seconds, air-dried, baked at 70°C for 1 hour, and stored at room temperature. FFPE blocks were sectioned on Vectabond-treated coverslips and stored in a coverslip storage box (CS-22, Qintay, LLC) at 4°C in a vacuum desiccator (Thermo Fisher) containing drierite desiccant (07-578-3A, Thermo Fisher) until use for CODEX experiments.

### CODEX

CODEX assays were performed as previously reported (12, 22) and as described below. Please see **Supplementary Table 3** for a complete description of buffers and solutions used.

#### CODEX Antibody DNA Conjugation

Maleimide-modified short DNA oligonucleotides (for sequences, refer to **Supplementary Table 1**) were purchased from TriLink. Oligonucleotides were first activated as previously described (12, 22). LTS filter tips (Rainin) and nuclease-free microcentrifuge tubes were used in the entire conjugation protocol to prevent contamination. Conjugations were performed with at least 100 µg of antibody per reaction at a 2:1 weight/weight ratio of oligonucleotide to antibody. Centrifugation steps were performed at 12,000 g for 8 min unless otherwise specified. Antibodies purchased were purified and carrier-free (for details on clones and manufacturers, refer to **Supplementary Table 1**). Antibodies were first loaded onto 50 kDa filters (UFC505096, Thermo Fisher) in microcentrifuge tubes and reduced using a mixture of 2.5 mM TCEP and 2.5 mM EDTA in PBS, pH 7.0, for 30 min at room temperature. Next, filter tubes were centrifuged and antibodies were washed with buffer C. Activated oligonucleotides were resuspended in buffer C containing NaCl at a final concentration of 400 mM. Activated oligonucleotides were then added to the concentrated, reduced, and washed antibodies and incubated for 2 hours at room temperature to allow conjugation to occur. Following this incubation, conjugated antibodies were washed three times in 450 µL of PBS containing 900 mM NaCl. Finally, conjugated antibodies were eluted by inverting filters, centrifuging at 3,000 g for 2 min, and diluted in PBS-based antibody stabilizer (nc0436689, Thermo Fisher) containing 0.5 M NaCl, 5 mM EDTA, and 0.02% w/v NaN3 (Sigma), and stored at 4°C.

#### CODEX FFPE Tissue Staining

Coverslips with 4-µm FFPE tissue sections were processed as described above for IHC. Briefly, tissue sections were deparaffinized by baking at 70°C for at least 1 h, followed by immersion in fresh xylene for 30 min (two separate containers, 15 min each). Sections were then rehydrated in descending concentrations of ethanol (twice in 100%, twice in 95%, once in 80%, once in 70%, twice in ddH2O; each step for 3 min). Coverslips were loaded into slide chambers and HIER was performed using a PT Link Pre-Treatment Module in 1X Target Retrieval Solution, pH9 (Agilent) at 97°C for 10 min. After antigen retrieval, slide chambers were removed from the module and allowed to equilibrate to room temperature for 30 min. Coverslips were washed twice for a total of 10 min in 1X TBS IHC wash buffer with Tween20 (Cell Marque). Sections were surrounded with a polyacrylamide gel (Bondic) to create a region for reagents to pool. Coverslips were blocked for 1 hour at room temperature using 100 µL of blocking buffer (S2 buffer with B1 (1:20), B2 (1:20), B3 (1:20), and BC4 (1:15)) to prevent non-specific antibody binding. DNA-conjugated antibody cocktails were prepared for each coverslip section. Antibodies were added to 50 µL of the blocking buffer and loaded onto a 50-kDa filter unit, concentrated by spinning at 12,000 g for 8 min and resuspended in the blocking buffer to a final volume of 100 µL. This antibody cocktail was then pipetted onto coverslip sections and incubated in a sealed humidity chamber overnight at 4°C on an orbital shaker for staining. Following overnight staining, coverslips were washed twice in buffer S2 for (2 minutes per wash) and fixed in buffer S4 containing 1.6% paraformaldehyde for 10 min. After fixation, coverslips were washed three times in 1X PBS. Coverslips were then incubated in 100% methanol on ice for 5 min, followed by three washes in 1X PBS. Fresh BS3 fixative solution was prepared immediately before final fixation by thawing and diluting one 15 µL aliquot of BS3 in 1 mL 1 X PBS. 200 µL of the BS3-PBS solution was added to coverslips and fixation was allowed to occur at room temperature for 20 min. Following this fixation step coverslips were washed three times in 1X PBS (using fresh PBS for each wash). All incubations and washes were performed in 6-well tissue culture plates (07-20083, Thermo Fisher). For an immediate multicycle and image acquisition, coverslips were placed in a coverslip glass container containing buffer H2. For a future multicycle and image acquisition, coverslips were stored in buffer S4 in a coverslip storage glass jar at 4°C for up to two weeks.

#### CODEX Image Acquisition

To create fluorescent oligonucleotide plates for multicycle rendering, appropriate fluorescent oligonucleotides (at a concentration of 400 nM) were aliquoted in wells of a black 96-well plate (07-200-762, Corning containing plate buffer (a mixture of buffer H2 plus Hoechst (62249, Thermo Fisher) nuclear stain (1:600) and 0.5 mg/ml sheared salmon sperm DNA). Details of the fluorescent oligonucleotides are provided in **Supplementary Table 1**. Each CODEX cycle contains up to 4 fluorescent channels (three for antibody visualization and one for nuclear stain). For each cycle, up to three fluorescent oligonucleotides (10 µL each) were added to 220 µL of plate buffer (containing Hoechst nuclear stain). For each empty channel, 10 µL of plate buffer was substituted for fluorescent oligonucleotides. A final well containing DRAQ5 (4084L, Cell Signalling Technology) (1:500 final dilution) was added as an additional nuclear stain. Plates were sealed with aluminum sealing film (14-222-342, Thermo Fisher) and kept at room temperature until use.

Chambers containing stained coverslips stored in buffer S4 were removed from 4°C and allowed to equilibrate to room temperature. Coverslips were then removed from S4 and placed in a separate chamber containing buffer H2. Coverslips were then removed from H2 and covered with a small piece of cling wrap. The exposed areas were washed with ddH2O to remove any residual salts and thoroughly dried using a vacuum aspirator. Coverslips were then mounted onto custom-made CODEX acrylic plates (Bayview Plastic Solutions) using double-sided clear adhesive tape (TMG-22, Qintay) to create a well for buffer exchange. A second layer of adhesive tape was added below the coverslip for additional leak protection. Next the cling wrap was removed from the section and the well was washed with H2. Nuclear staining was performed by adding to the well Hoechst nuclear stain at a dilution of 1:1000 in H2 buffer for 30s followed by three washes with buffer H2. The CODEX acrylic plate was mounted onto a custom-designed plate holder and securely tightened onto the stage of a Keyence BZ-X710 inverted fluorescence microscope.

Cycles of hybridization, buffer exchange, image acquisition, and stripping were then performed using an Akoya CODEX EA (early access) instrument and CODEX Driver EA2 (version 2.0.0.29). Briefly, that instrument performs hybridization of the fluorescent oligonucleotides in a hybridization buffer, imaging of tissues in buffer H2, and stripping of fluorescent oligonucleotides in the stripping buffer.

CODEX multicycle automated imaging of regions of interest or TMA cores was performed using a CFI Plan Apo 20x/0.75 objective (Nikon). The multipoint function of the BZ-X viewer software (BZ-X ver. 1.3.2, Keyence) was manually programmed to align with the center of each TMA core and set to 25 Z stacks. Nuclear stain (Hoechst, 1:3000 final concentration) was imaged in each cycle at an exposure time of roughly 8 ms. DRAQ5 nuclear stain was visualized in the last imaging cycle at an exposure time of roughly 118 ms. The respective channels were imaged in the automated run using pre-determined optimized exposure times (See **Supplementary Table 1**).

#### CODEX Computational Image Processing and Analysis

Raw TIFF images produced during image acquisition were processed using the CODEX Toolkit uploader [Version 1.5.5 (6)]. The toolkit uploader concatenates Z-stack images and performs drift compensation based on alignment of Hoechst nuclear stain across images. It also removes the out-of-focus light using the Microvolution deconvolution algorithm (Microvolution) and subtracts the background using blank imaging cycles without fluorescent oligonucleotides. It finally creates hyperstacks of all fluorescence channels and imaging cycles of the imaged regions. Hyperstacks were loaded and visualized on FIJI version 2.0.0 (30). Antibody staining performance was visually inspected across each channel and cycle using two and four-color overlays. Segmentation was performed using the nuclear channel, and cell features extracted as previously described (6).

#### Cell type Annotation and Differential Marker Analysis

Cell populations were gated as follows. All nucleated cells were first identified by a positive nuclear signal. Granulocytes (CD66+CD45+) and non-granulocyte (CD66-CD45+) immune cells were first subsetted. From the non-granulocytes, CD4 (CD3+, CD4+) and CD8 (CD3+, CD8+) T cells, B cells (CD3-, CD20+, CD21+), macrophages (CD3-, CD20-, CD21-, CD68+, CD163+), NK cells (CD3-, CD20-, CD21-, CD68-, CD163-, CD56+), dendritic cells (CD3-, CD20-, CD21-, CD68-, CD163-, CD209+), and plasma cells (CD3-, CD20-, CD21-, CD68-, CD163-, CD138+) populations were gated. A heatmap was then constructed showing the median marker expression (z-score) across cell populations.

## Data Availability Statement

The raw data supporting the conclusions of this article will be made available by the corresponding author without undue reservation.

## Ethics Statement

The animal study was reviewed and approved by NIAID Division of Clinical Research Animal Care and Use Committee and the Institutional Animal Care and Use Committee of the University of California, Davis.

## Author Contributions

Conceptualization: LH, GN, and DM. Generated samples: RB, JL, BD-K, JK, RA, PC, RR, RK, KR, DL, and LH. Validated reagents and tools: SJ, NM, EM, AD, JG, and IZ. Conducted experiments: SJ, NM, HC, and DM. Analyzed data: SJ, NM, HC, DP, CS, YG, JH, XL, GN, and DM. Writing: SJ, NM, HC, XL, LH, GN, and DM. Supervision: LH, GN, and DM. Funding Acquisition: GN and DM. Co-first authors reserve the right to place their names first in their CV. All authors contributed to the article and approved the submitted version.

## Funding

This work was supported in part by the U.S. Food and Drug Administration (FDA) Medical Countermeasures Initiative contracts 75F40120C00176 and HHSF223201610018C (DM and GN); Defence Science and Technology Laboratory (DSTL) DSTL/AGR/00980/01 (GN); The Rachford and Carlota A. Harris Endowed Professorship (GN); The Bill and Melinda Gates Foundation OPP1113682 (GN) and INV-002704 (SJ and GN); Battelle Memorial Institute’s former prime contract with the US National Institute of Allergy and Infectious Diseases (NIAID) under Contract No. HHSN272200700016I and Laulima Government Solutions, LLC’s current prime contract with NIAID under Contract No. HHSN272201800013C; Office of Research Infrastructure Program, Office of The Director, National Institutes of Health (P51OD011107; CNPRC). JH was supported by an NIH T32 Fellowship (T32CA196585), and an American Cancer Society-Roaring Fork Valley Postdoctoral Fellowship (PF-20-032-01-CSM). EM was supported by the NSF Graduate Research Fellowship (grant no. 2017242837) and NIH 5T32AI007290. The article reflects the views of the authors and does not represent views or policies of the FDA, DSTL, NIH, or any other funding sources listed here. All funders were not involved in the study design, collection, analysis, interpretation of data, the writing of this article or the decision to submit it for publication.

## Conflict of Interest

GN and YG are co-founders and stockholders of Akoya Biosciences, Inc. GN, YG, and DM are inventors on CODEX-related patents US9909167 and US9909167B2, respectively. CS is a scientific advisor to Enable Medicine, Inc. CS, DP, and GN are inventors on a patent application related to CODEX (PCT/US2021/016641). EFM has previously consulted for Ionpath, Inc.

The remaining authors declare that the research was conducted in the absence of any commercial or financial relationships that could be construed as a potential conflict of interest.

## Publisher’s Note

All claims expressed in this article are solely those of the authors and do not necessarily represent those of their affiliated organizations, or those of the publisher, the editors and the reviewers. Any product that may be evaluated in this article, or claim that may be made by its manufacturer, is not guaranteed or endorsed by the publisher.

## Supplementary Material

Supplementary Figure 1 Immunohistochemistry validation of antibodies on human and rhesus immune tissues. Representative IHC images for indicated markers on human (left) and rhesus (right) tissues. Spleen tissue is shown unless otherwise indicated. *human spleen, rhesus bone marrow; ** human tonsil, rhesus lymph node.

Supplementary Table 1 Antibody panel information. Antibody, clone, supplier, product number, oligonucleotide number, oligonucleotide sequence, oligonucleotide channel, imaging order.

Supplementary Table 2 All antibodies tested in this study. Antibody, clone, Manufacturer reported reactivity, species, company, catalog number, manufacturer recommendation for IHC, dilutions tested for IHC, pass/fail of antibody in our validation pipeline.

Supplementary Table 3 Composition of buffers and reagents for CODEX.
