# Supplementary figures and images for: Rhesus Macaque CODEX Multiplexed Immunohistochemistry Panel for Studying Immune Responses During Ebola Infection
Source: Front Immunol. 2021 Dec 6;12:729845. doi: 10.3389/fimmu.2021.729845 (PMC8685521; doi:10.3389/fimmu.2021.729845)

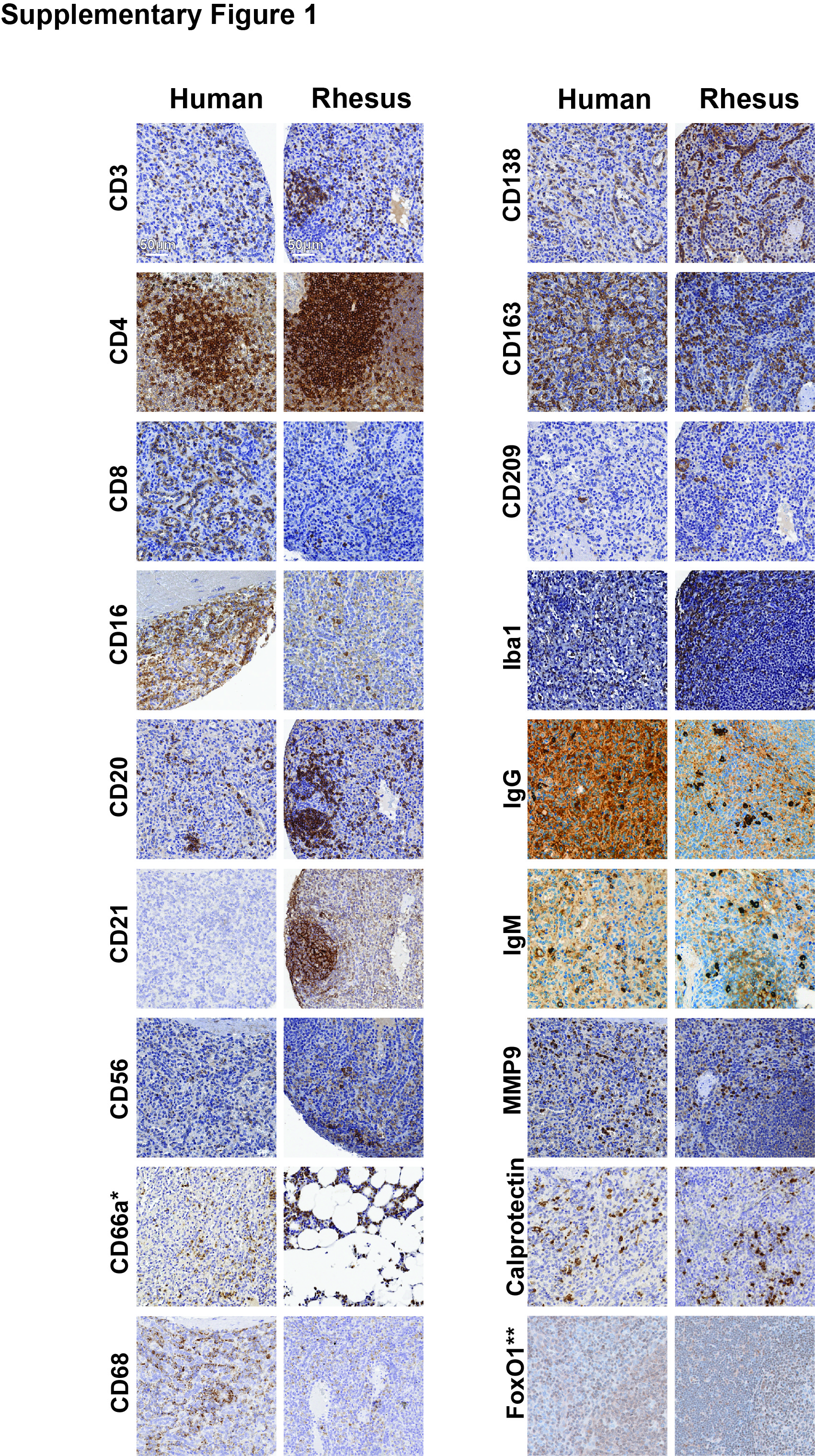

Supplement: Supplementary Figure 1 — Immunohistochemistry validation of antibodies on human and rhesus immune tissues. Representative IHC images for indicated markers on human (left) and rhesus (right) tissues. Spleen tissue is shown unless otherwise indicated. *human spleen, rhesus bone marrow; ** human tonsil, rhesus lymph node. [file Image_1.jpg]
